# Fatigue nach einer COVID-19-Erkrankung im Zusammenhang mit Depressivität und Ängstlichkeit bei Versicherten aus Gesundheits- und Sozialberufen

**DOI:** 10.1007/s00103-024-03953-y

**Published:** 2024-09-27

**Authors:** Svenja Mertens, Albert Nienhaus, Claudia Peters, Uwe Koch-Gromus

**Affiliations:** 1 Institut und Poliklinik für Medizinische Psychologie, Universitätsklinikum Hamburg-Eppendorf, Martinistr. 52, 20246 Hamburg, Deutschland; 2 Institut für Versorgungsforschung in der Dermatologie und bei Pflegeberufen (IVDP), Universitätsklinikum Hamburg-Eppendorf, Hamburg, Deutschland

**Keywords:** Post-Covid-Syndrom, Postvirale Fatigue, Depressivität, COVID-19, Gesundheitsberufe, Post-COVID syndrome, Post-viral fatigue, Depression, COVID-19, Healthcare workers

## Abstract

**Hintergrund:**

Fatigue bezeichnet einen andauernden Erschöpfungszustand, der auf eine Infektionskrankheit folgen kann. Sie zählt zu den häufigsten Symptomen beim Post-Covid-Syndrom (PCS). Auch bei psychischen Erkrankungen kann Fatigue auftreten, allerdings ist Fatigue, wie andere chronische Erkrankungen, auch selbst ein Risikofaktor für Depressivität und Ängstlichkeit. Ziel der Analyse ist es, zu untersuchen, inwieweit sich Fatigue und Depressivität/Ängstlichkeit gegenseitig bedingen und ob es Unterschiede zwischen PCS-Betroffenen und vollständig Genesenen gibt.

**Methodik:**

In einer Längsschnittuntersuchung mit 3 Messzeitpunkten wurden Versicherte der Berufsgenossenschaft für Gesundheitsdienst und Wohlfahrtspflege, die im Jahr 2020 mit SARS-CoV‑2 infiziert waren, zu Fatigue, Depressivität/Ängstlichkeit und lang anhaltenden COVID-19-Symptomen befragt. Zur Analyse der längsschnittlichen Effekte der beiden Variablen wurde ein kreuzlagiges Paneldatenmodell angewandt.

**Ergebnisse:**

Die Stichprobe (*n* = 860) weist zu den 3 Messzeitpunkten einen Anteil von 68,7–75,1 % an PCS-Betroffenen auf. Das Modell zeigt eine Güte von R^2^ = 61,49 % und durchgehend signifikante Effekte, jedoch unterscheiden sich die kreuzlagigen Pfade nicht signifikant voneinander. Wird danach stratifiziert, ob eine PCS-Symptomatik vorliegt, schwächen sich in beiden Gruppen die kreuzlagigen Effekte ab, während nur in der Gruppe der PCS-Betroffenen die Effekte von Fatigue auf Depressivität und Ängstlichkeit bestehen bleiben.

**Diskussion:**

Die vorliegenden Ergebnisse zeigen einen wechselseitigen Zusammenhang von Fatigue- und Depressivitäts‑/Ängstlichkeitssymptomatik. PCS-Betroffene könnten von psychotherapeutischen Behandlungen aufgrund ihrer Fatigue profitieren, da dem Entstehen von Depressionen oder Angststörungen vorgebeugt werden kann.

## Abstract (en)

**Background:**

Fatigue is one of the most frequent symptoms in post-COVID syndrome (PCS) patients and describes a long-term feeling of tiredness and exhaustion. Frequently, it follows an infection or is a component of depressive symptoms. But fatigue itself is also a risk to mental health like other chronic conditions. The objective of this paper was to analyse the relationship between fatigue and depression/anxiety and the differences between PCS patients and fully recovered COVID-19 cases.

**Methods:**

In a longitudinal study with three measurement points, insured members of the Statutory Accident Insurance and Prevention in the Health and Welfare Service with a SARS-CoV‑2 infection in 2020 were asked about fatigue, depression/anxiety and persisting COVID-19 symptoms. To analyse the longitudinal effects of the two variables, a cross-lagged panel model was applied.

**Results:**

In the present sample (*n* = 860), a proportion of 68.7% to 75.1% of people was suffering from PCS. The results showed a model fit of R^2^ = 61.49% and all effects were significant, but the cross-lagged effects were not significantly different from each other. If stratified according to whether PCS symptoms were present, the cross-lagged effects weakened in both groups, while the effects of fatigue on depression and anxiety only remained in the group of those affected by PCS.

**Discussion:**

The results show a relationship in both directions between fatigue and depression/anxiety. PCS patients can benefit from psychotherapeutic treatment to tackle fatigue and prevent depression and anxiety.

## Einleitung

Das Virus SARS-CoV‑2 führte bis heute zu über 775 Mio. Infektionen weltweit [1]. Während die Hospitalisierungsraten und intensivmedizinischen Behandlungen aufgrund von akuten COVID-19-Erkrankungen zurückgehen [2], besteht weiterhin das Problem von lang anhaltenden Symptomen nach der Akuterkrankungsphase. Bleiben diese über einen Zeitraum von 3 Monaten nach Ende der Akutphase bestehen oder treten neue Symptome auf, die über mindestens 2 Monate hinweg bestehen, wird dies als Post-Covid-Syndrom (PCS) bezeichnet. Dabei können die Symptome sowohl seit der akuten Phase bestehen als auch im weiteren Verlauf neu auftreten [3]. Die Prävalenz von lang anhaltenden Symptomen liegt je nach Studie bei ungefähr 2–15 % in der Allgemeinbevölkerung [4, 5] und 54 % bei während der Akuterkrankungsphase stationär behandelten Personen [6]. Zu den häufigsten Symptomen bei PCS zählen Erschöpfung und Müdigkeit (auch als „Fatigue“ bezeichnet), Kopfschmerzen und Konzentrationsschwierigkeiten [7]. PCS-Betroffene zeigen zudem häufig erhöhte Werte von Depressivität und Ängstlichkeit [8].

Persistierenden Erschöpfungszustände sind nicht erst seit der COVID-19-Pandemie bekannt. Das chronische Fatigue-Syndrom (CFS) oder auch myalgische Encephalomyelitis (ME) genannt zeichnet sich durch eine mindestens 6 Monate anhaltende Erschöpfung aus, die sich nach kognitiven oder körperlichen Belastungen deutlich verstärkt. Zudem leiden Betroffene häufig unter Schmerzen, Konzentrationsstörungen, Schlafproblemen und Verdauungsbeschwerden [9]. Meistens entsteht die CFS/ME-Symptomatik nach Infektionserkrankungen [10], jedoch ist die Symptomatik diffus und kann meist nicht mit standardisierten Testverfahren diagnostiziert werden [11]. Psychische Erkrankungen tragen auch zu einer Entwicklung von CFS/ME bei, jedoch lässt sich auch der umgekehrte Effekt beobachten, dass sich depressive Symptome bei CFS/ME-Betroffenen ähnlich häufig entwickeln wie bei anderen einschränkenden chronischen Erkrankungen [12]. In einer Schweizer Pilotstudie berichteten CFS/ME-Betroffene, dass sie Gefühle der Depression, Angst, Hoffnungslosigkeit und Traurigkeit im Zusammenhang mit ihrer Erkrankung verspüren. Ein Großteil derer, die von Depressionen berichteten, entwickelte diese erst nach Auftreten des Fatigue-Syndroms [13].

Bei der Behandlung des PCS wird häufig mit Atemtherapie und individuell angepasstem Kraft- und Muskelausdauertraining zur Wiederherstellung der körperlichen Leistungsfähigkeit gearbeitet, teilweise ergänzt mit Psycho- oder Ergotherapie [14]. Bisherige Evaluationsstudien zu Rehabilitationsangeboten bei PCS zeigen einen positiven Effekt auf die Fatigue sowie auf Depressivität und Ängstlichkeit, jedoch wird das Niveau an Leistungsfähigkeit vor der COVID-19-Erkrankung nicht wieder erreicht [15, 16]. In einem systematischen Review von Behandlungsmethoden wurde gezeigt, dass wirksame Ansätze interdisziplinär gestaltet und nicht nur auf einen Faktor, wie bspw. die Wiederherstellung der körperlichen Leistungsfähigkeit, fokussiert sein sollten [17]. Es besteht dementsprechend weiterer Forschungsbedarf für geeignete Behandlungsmöglichkeiten von PCS.

Eine besondere Risikogruppe für PCS, die auch in der vorliegenden Arbeit betrachtet wird, sind Personen, die im Gesundheits- und Sozialwesen beschäftigt sind, da ihr SARS-CoV-2-Infektionsrisiko verglichen mit Personen aus anderen Sektoren doppelt so hoch war [18, 19]. Die Berufsgenossenschaft für Gesundheitsdienst und Wohlfahrtspflege (BGW) versichert im Gesundheits- und Sozialbereich Beschäftigte in Form einer Unfallversicherung. Aufgrund der erhöhten Infektionszahlen und dementsprechend großen Zahl an PCS-Betroffenen besteht ein gesteigertes Interesse der BGW, sowohl Erkrankungsmechanismen und Symptomatik der akuten COVID-19-Erkrankung und PCS zu untersuchen als auch geeignete Behandlungsmöglichkeiten zu finden und zu evaluieren. Deshalb initiierte die BGW eine Längsschnittstudie, die das Ziel hat, den langfristigen Verlauf einer COVID-19-Erkrankung zu verfolgen.

Zum ersten Messzeitpunkt dieser Längsschnittstudie wurde bei den Versicherten ein querschnittlicher Zusammenhang zwischen der Schwere von Fatigue, Depressivität und Ängstlichkeit festgestellt. Je stärker die Fatigue ausgeprägt war, desto höhere Werte ergaben sich für Depressivität und Ängstlichkeit. Zudem wurde bei dieser Auswertung herausgefunden, dass psychische Vorerkrankungen einen Prädiktor für höhere Fatigue-Werte bei den ehemals Infizierten darstellen [20]. Allerdings bleibt unklar, inwiefern es längsschnittliche Effekte zwischen diesen beiden Variablen gibt, wenn keine psychische Vorerkrankung vorliegt. Bisher wird Fatigue häufig als Symptom von depressiven Erkrankungen aufgeführt [21], jedoch ist es bezüglich geeigneter Interventionen auch wichtig zu analysieren, inwieweit die ggf. im Zusammenhang mit PCS entstandene Fatigue-Symptomatik zu Depressivität und Ängstlichkeit führen kann, sodass dadurch ein Bedarf an psychotherapeutischen Angeboten bei PCS entstehen könnte.

Vorrangiges Ziel dieser Arbeit ist, zu untersuchen, welche Vorhersagekraft Fatigue- und Depressivitäts- und Ängstlichkeitssymptomatik für die jeweils andere Variable zum jeweils nächsten Messzeitpunkt haben. Zudem wird analysiert, ob es diesbezüglich Unterschiede zwischen Personen mit und ohne PCS-Symptomatik gibt.

## Methoden

Die Datengrundlage für diese Studie stammt aus einer Längsschnitterhebung der BGW, für die im Februar 2021 alle 4325 Versicherten aus den Bezirksverwaltungen Köln und Dresden, die im Jahr 2020 mit dem Virus SARS-CoV‑2 infiziert waren, kontaktiert und nach schriftlicher Einwilligung mittels Papierfragebogen befragt wurden. Neben demografischen Charakteristika beinhaltete dieser unter anderem Fragen zur akuten und lang anhaltenden Symptomatik und speziell zu Fatigue, Depressivität und Ängstlichkeit. Bezüglich der lang anhaltenden Symptomatik wurden die Versicherten zu jedem der 3 Messzeitpunkte (Februar 2021, Oktober 2021, März 2022) gefragt, ob sie noch an Symptomen der vergangenen akuten COVID-19-Erkrankung leiden. Diese Frage konnte bejaht oder verneint werden.

Die Fatigue-Symptomatik wurde mittels der Subskala „Allgemeine Erschöpfung“ des Multidimensional Fatigue Inventory (MFI) erhoben. Diese umfasst 4 Items, die auf einer 5‑stufigen Likert-Skala (1 „überhaupt nicht“ bis 5 „maximal“) erhoben und aufsummiert werden. Der Score kann Werte zwischen 4 und 20 annehmen. Als klinisch relevant werden Werte ab 16 Punkten kategorisiert [22]. Für diese Subskala wurde eine interne Konsistenz von *α* = 0,76 und eine Konvergenzvalidität von $\rho$= 0,70 berechnet, was für eine gute Reliabilität und Validität spricht [23].

Depressivität und Ängstlichkeit wurden mittels Patient Health Questionnaire for Depression and Anxiety‑4 (PHQ-4) erhoben. Der Fragebogen besteht aus jeweils 2 Items zu Ängstlichkeit und Depressionen. Diese beiden Subskalen können jeweils Werte von 0 bis 6 Punkten annehmen, die Gesamtskala zeigt dementsprechend Werte von 0 bis 12 Punkten. Als klinisch auffällig werden Werte von über 2 auf einer oder beiden Subskalen gewertet [24]. Auch dieses Instrument zeigt eine hohe Reliabilität von $\Upomega$= 0,85 und eine gute Konstruktvalidität von r = 0,9 [25].

Im Oktober 2021 und im März 2022 wurden die Versicherten noch weitere Male kontaktiert und zu denselben Symptomen befragt. Eine Auswertung zum ersten Messzeitpunkt mit 2052 Teilnehmenden wurde schon im Jahr 2022 im Bundesgesundheitsblatt veröffentlicht und erbrachte unter anderem die in der Einleitung dargestellten Ergebnisse [20].

Es wurden nur Versicherte in die Auswertung einbezogen, zu denen alle Angaben zu den hier relevanten Variablen bei allen 3 Messzeitpunkten vorlagen und die vor ihrer Infektion keine selbst oder von ärztlicher Seite diagnostizierten psychischen Beeinträchtigungen aufwiesen, um Verzerrungen zu vermeiden. Zur Deskription der vorliegenden Variablen wurden Mittelwerte und Standardabweichungen sowie die relativen Häufigkeiten der Personen, die auffällige bzw. klinisch relevante Werte aufwiesen, berichtet. Vorwegzunehmen ist, dass die Variablen nicht normalverteilt sind. Da die Stichprobe jedoch sehr groß und die mögliche Spannweite der Variablen gering ist, wurde zur Beschreibung der Mittelwert dem Median vorgezogen. Um die Gruppen mit und ohne PCS-Symptome zu vergleichen, wurden T‑Tests und Chi-Quadrat (ggf. mit Fisher-Exact-Signifikanzanpassung) berechnet.

**Abb. 1:**
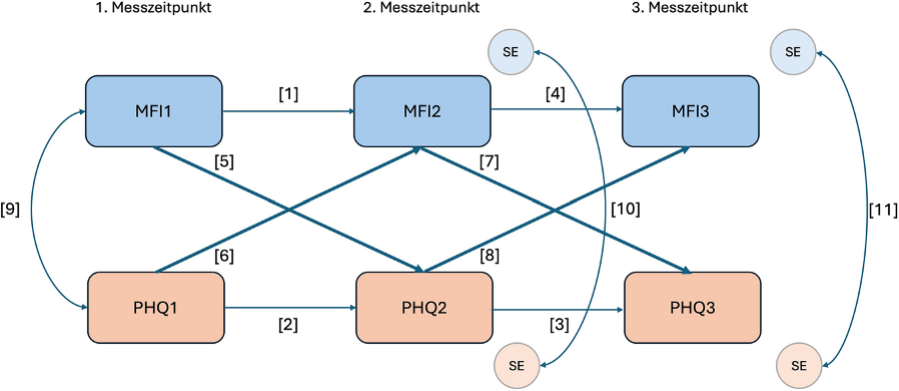
Methodik des kreuzlagigen Paneldatenmodells (CLPM; eigene Abbildung). MFI1-3: Fatigue zu den jeweiligen Messzeitpunkten, PHQ 1–3: Depressivität und Ängstlichkeit zu den jeweiligen Messzeitpunkten. Nr. 1–4: Autoregressive Effekte (Zusammenhänge mit derselben Variable von einem zum nächsten Messzeitpunkt), Nr. 5–8: kreuzlagige Effekte (Zusammenhänge mit der jeweils anderen Variable von einem zum nächsten Messzeitpunkt), Nr. 9: Kovarianz zum ersten Messzeitpunkt, Nr. 10: Kovarianz der Standardfehler zum zweiten Messzeitpunkt, Nr. 11: Kovarianz der Standardfehler zum dritten Messzeitpunkt

In der vorliegenden Auswertung wurde anschließend anhand eines Strukturgleichungsmodells getestet, inwiefern es Zusammenhänge zwischen den beiden gemessenen Variablen Fatigue und Depressivität/Ängstlichkeit zu den verschiedenen Zeitpunkten gab. Mit dem kreuzlagigen Paneldatenmodell (Cross Lagged Panel Model, CLPM) wurden längsschnittlich erfasste Daten analysiert und dabei die autoregressiven Effekte der einzelnen Variablen über die Zeit berücksichtigt (Abb. 1, Nr. 1–4). So können in diesem Fall die Beziehungen zwischen den Ergebnissen des MFI und des PHQ‑4 analysiert werden, während gleichzeitig berücksichtigt wird, dass diese beiden Variablen sich über die Zeit verändern können, ohne dass dies mit der jeweils anderen Variable assoziiert ist.

Im Fokus des CLPM stehen die kreuzlagigen, d. h. sich überkreuzenden Effekte (Abb. 1, Nr. 5–8). Hier wird die Vorhersagekraft der einen Variable für die andere Variable zum nächsten Messzeitpunkt gemessen [26]. Laut Orth et al. [27] sind Effekte ab $\upbeta$= 0,03 als kleine, ab 0,07 als mittlere und ab 0,11 als starke Effektstärken einzuordnen. Damit von Kausalität ausgegangen werden kann, müssen sich die beiden kreuzlagigen Effekte signifikant unterscheiden [26]. Zudem werden die Kovarianzen zum ersten Messzeitpunkt (Nr. 9) und die Kovarianzen der Standardfehler zum zweiten (Nr. 10) und dritten Messzeitpunkt (Nr. 11) getestet, um abzuschätzen, inwiefern andere Faktoren auf die beiden vorliegenden Variablen wirken. Die Modellgüte wird außerdem mittels R‑Quadrat abgeschätzt. Da die beiden Variablen durch den PHQ‑4 und den MFI nicht mit derselben Skala gemessen werden, werden durchgängig standardisierte Koeffizienten genutzt.

Um Unterschiede zwischen Personen mit PCS-Symptomen und vollständig Genesenen sichtbar zu machen, wurde danach stratifiziert, ob die Versicherten zum zweiten Messzeitpunkt angaben, weiterhin unter lang anhaltenden Symptomen der COVID-19-Erkrankung zu leiden, da der zweite Messzeitpunkt in alle autoregressiven und kreuzlagigen Effekte miteinbezogen wird.

Die deskriptiven Analysen wurden mit SPSS Version 29 (IBM, Armonk, NY, USA), die Analysen zum Strukturgleichungsmodell mit STATA Version 18.0 (StataCorp LLC, College Station, TX, USA) durchgeführt.

## Ergebnisse

### Stichprobe

**Abb. 2:**
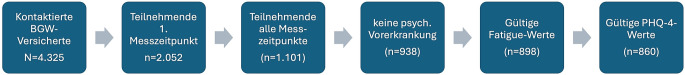
Stufenweise Filterung der Stichprobe (eigene Abbildung). *BGW* Berufsgenossenschaft für Gesundheitsdienst und Wohlfahrtspflege, *PHQ‑4* Patient Health Questionnaire for Depression and Anxiety‑4

Von den initial 2052 rekrutierten Personen konnten nach den oben genannten Kriterien *n* = 860 Personen in die Auswertung eingeschlossen werden (Abb. 2), von denen sich *n* = 703 (81,7 %) dem weiblichen Geschlecht zugehörig fühlten. Das durchschnittliche Alter lag bei MW = 49,2 (SD = 11,9) Jahren bei einer Spannweite von 18 bis 81 Jahren. Zum ersten Messzeitpunkt wurde auch die berufliche Tätigkeit abgefragt. Hier gaben *n* = 505 (58,7 %) Personen an, einer pflegerischen Tätigkeit nachzugehen. Insgesamt *n* = 100 (11,6 %) waren ärztlich und *n* = 56 (6,5 %) therapeutisch tätig. Nicht erwerbstätig oder bereits berentet waren *n* = 11 (1,3 %) Personen. Die übrigen *n* = 188 (21,8 %) Personen waren in verschiedenen Bereichen tätig, dazu gehören z. B. pädagogische Tätigkeiten, Assistenzen, Leitungen und Servicekräfte. Die Ergebnisse für die Untergruppen mit und ohne PCS zum zweiten Messzeitpunkt sind in Tab. 1 dargestellt.

**Tab. 1:** Ergebnisse des MFI (Multidimensional Fatigue Inventory) und des PHQ‑4 (Patient Health Questionnaire for Depression and Anxiety-4) insgesamt und stratifiziert nach Vorliegen der Symptomatik eines Post-Covid-Syndroms (PCS)

		Gesamt	Mit PCS	Ohne PCS
Soziodemografische Merkmale	*Geschlecht (n (%))*			
	Weiblich	703 (81,7)	527 (86,0)	176 (71,3)
	Männlich	157 (18,3)	86 (14,0)	71 (28,7)
	*Alter (MW (SD))*	49,2 (11,9)	49,9 (11,3)	47,4 (12,9)
	*Tätigkeit (n (%))*			
	Pflegerisch	505 (58,7)	384 (62,6)	121 (49,0)
	Ärztlich	100 (11,6)	41 (6,7)	59 (23,9)
	Therapeutisch	56 (6,5)	42 (6,9)	14 (5,7)
	Andere	188 (21,8)	146 (23,8)	53 (21,4)
1. Messzeitpunkt	*n (%)*	860 (100)	646 (75,1)	214 (24,9)
	*MFI (MW (SD))*	10,9 (3,8)	11,9 (3,4)	7,8 (3,2)
	*PHQ (MW (SD))*	2,5 (2,5)	2,9 (2,6)	1,1 (1,4)
	*Depressivität (n (%))*	128 (14,9)	122 (18,9)	6 (2,8)
	*Ängstlichkeit (n (%))*	126 (14,7)	121 (18,7)	5 (2,3)
	*Fatigue (n (%))*	82 (9,5)	81 (12,5)	1 (0,5)
2. Messzeitpunkt	*n (%)*	860 (100)	613 (71,3)	247 (28,7)
	*MFI (MW (SD))*	11,4 (3,3)	12,4 (2,8)	8,7 (3,0)
	*PHQ (MW (SD))*	2,6 (2,6)	3,1 (2,6)	1,3 (2,0)
	*Depressivität (n (%))*	132 (15,3)	122 (19,9)	10 (4,0)
	*Ängstlichkeit (n (%))*	120 (14,0)	109 (17,8)	11 (4,5)
	*Fatigue (n (%))*	82 (9,5)	74 (12,1)	1 (0,4)
3. Messzeitpunkt	*n (%)*	860 (100)	591 (68,7)	269 (31,3)
	*MFI (MW (SD))*	11,3 (3,3)	12,5 (2,8)	8,8 (3,0)
	*PHQ (MW (SD))*	2,6 (2,6)	3,2 (2,7)	1,3 (1,7)
	*Depressivität (n (%))*	135 (15,7)	130 (22,0)	5 (1,9)
	*Ängstlichkeit (n (%))*	135 (15,7)	122 (20,6)	13 (4,8)
	*Fatigue (n (%))*	75 (8,7)	81 (13,7)	1 (0,4)

Abkürzungen: *MW* Mittelwert, *SD* Standardabweichung

### Fatigue-Symptomatik

In Tab. 1 sind die wichtigsten Werte zu Fatigue, Depressivität und Ängstlichkeit dargestellt. Der Mittelwert der Fatigue-Symptomatik bleibt über die 3 Messzeitpunkte relativ konstant. Zum ersten Messzeitpunkt beträgt er MW = 10,9 (SD = 3,8), zum zweiten ZeitpunktMW = 11,4 (SD = 3,3) und zum dritten MW = 11,3 (SD = 3,3). Die mögliche Spanne von 4 bis 20 Punkten war durchgängig voll ausgeschöpft. Klinisch relevante Fatigue-Werte (MFI ≥ 16) wurden zum ersten und zum dritten Messzeitpunkt jeweils bei *n* = 82 (9,5 %) Personen gemessen. Zum zweiten Messzeitpunkt ist der Anteil bei 8,7 % (entspricht *n* = 75 Personen).

Wird danach stratifiziert, ob die Teilnehmenden zum jeweiligen Messzeitpunkt weiterhin PCS-Symptome berichteten, werden zu allen Messzeitpunkten signifikante Gruppenunterschiede (*p* < 0,001) im Mittelwert der Fatigue-Werte und in der Häufigkeit der klinisch relevanten Ergebnisse sichtbar.

Zum ersten Messzeitpunkt hat die Gruppe mit PCS-Symptomen (*n* = 646; 75,1 %) einen Mittelwert von MW = 11,9 (SD = 3,4), während die Gruppe ohne lang anhaltende Symptome (*n* = 214; 24,9 %) einen Mittelwert von MW = 7,8 (SD = 3,2) aufweist. Das Maximum liegt in dieser Gruppe bei 16, welches bei einer Person gemessen wurde. Dementsprechend ist dies auch die einzige Person mit einem klinisch relevanten Ergebnis in dieser Gruppe. In der Gruppe mit PCS haben *n* = 81 (12,5 %) Personen ein auffälliges Ergebnis.

Zum zweiten Messzeitpunkt zeigt sich ein Mittelwert von MW = 8,7 (SD = 3,0) in der Gruppe ohne PCS (*n* = 247; 28,7 %) und von MW = 12,4 (SD = 2,8) in der Gruppe mit PCS (*n* = 613; 71,3 %). Auch hier ist es lediglich eine Person, die keine PCS-Symptome, aber ein klinisch relevantes Ergebnis im MFI-Score zeigt, zu diesem Messzeitpunkt liegt dieses Ergebnis bei 18. In der Gruppe mit lang anhaltenden Symptomen zeigen *n* = 74 (12,1 %) klinisch auffällige Ergebnisse.

Auch zum dritten Messzeitpunkt gibt es nur minimal abweichende Ergebnisse. Die Mittelwerte des MFI lagen hier bei MW = 8,8 (SD = 3,0) in der Gruppe ohne Symptome (*n* = 269; 31,3 %) und bei MW = 12,5 (SD = 2,8) in der Gruppe mit anhaltenden Symptomen (*n* = 591; 68,7 %). Wieder gab es eine Person in der Gruppe ohne Symptome mit einem Wert von 16, der als klinisch auffällig kategorisiert wird. In der Gruppe mit Symptomen waren es wie zum ersten Messzeitpunkt *n* = 81 (13,7 %).

### Depressivität und Ängstlichkeit

Bei Betrachtung der Werte für Depressivität und Ängstlichkeit zeigt sich in der Gesamtgruppe zu allen Messzeitpunkten ein ähnlicher Mittelwert. Zum ersten Messzeitpunkt liegt dieser bei MW = 2,5 (SD = 2,5), zum zweiten und dritten Messzeitpunkt jeweils bei MW = 2,6 (SD = 2,6). Es wurde zu allen Zeitpunkten die mögliche Spanne von 0 bis 12 angegeben. Auffällige Ergebnisse wurden bei *n* = 128 (14,9 %) Personen zu Depression und bei *n* = 126 (14,7 %) zu Ängstlichkeit gemessen. Zum zweiten Messzeitpunkt sind *n* = 132 (15,3 %) der Ergebnisse zu Depressivität und *n* = 120 (14,0 %) der Ergebnisse zu Ängstlichkeit auffällig. Beim letzten Messzeitpunkte wurden jeweils *n* = 135 (15,7 %) auffällige Werte zu Depressivität und Ängstlichkeit gemessen.

Beim Vergleich der Gruppen mit und ohne PCS-Symptome zeigen sich zu allen Messzeitpunkten signifikante Gruppenunterschiede im Mittelwert und in der Häufigkeit auffälliger Werte in Depressivität und Ängstlichkeit (*p* < 0,001).

Zum ersten Messzeitpunkt liegt der Mittelwert in der Gruppe ohne PCS bei MW = 1,1 (SD = 1,4) und das Maximum bei x = 6. Hier lagen bei *n* = 6 (2,8 %) auffällige Werte zu Depressionen und bei *n* = 5 (2,3 %) auffällige Werte zu Ängstlichkeit vor. In der Gruppe mit PCS liegt der Mittelwert bei MW = 2,9 (SD = 2,6). Zu Depressionen lagen bei *n* = 122 (18,9 %) und zu Ängstlichkeit bei *n* = 121 (18,7 %) auffällige Werte vor.

Zum zweiten Messzeitpunkt zeigt sich, dass auch hier die Gruppe mit PCS einen höheren Mittelwert von MW = 3,1 (SD = 2,6) als die Gruppe ohne PCS (MW = 1,3; SD = 2,0) hatte, jedoch zeigte sich die volle Spannweite von 0 bis 12 in beiden Gruppen. Weiterhin lagen in der Gruppe mit PCS bei *n* = 122 (19,9 %) auffällige Werte zu Depressivität und *n* = 109 (17,8 %) auffällige Werte zu Ängstlichkeit vor. Bei den Personen ohne PCS-Symptome haben *n* = 10 (4,0 %) erhöhte Werte bei Depressivität und *n* = 11 (4,5 %) bei Ängstlichkeit.

Die Werte zum dritten Messzeitpunkt weichen mit MW = 3,2 (SD = 2,7) in der Gruppe mit PCS-Symptomen und mit MW = 1,3 (SD = 1,7) in der Gruppe ohne PCS-Symptome nur geringfügig vom zweiten Messzeitpunkt ab. Allerdings reicht hier die Spannweite der Gruppe ohne Symptome lediglich von 0 bis 8. Insgesamt *n* = 5 (1,9 %) Personen ohne PCS zeigen auffällige Werte zu Depressivität und *n* = 13 (4,8 %) auffällige Werte zu Ängstlichkeit. In der Gruppe der Personen mit PCS-Symptomen wurde bei *n* = 130 (22,0 %) Personen ein erhöhter Wert zu Depressivität und bei *n* = 122 (20,6 %) ein erhöhter Wert zu Ängstlichkeit gemessen.

### Auswertung des kreuzlagigen Paneldatenmodells (CLPM)

**Abb. 3:**
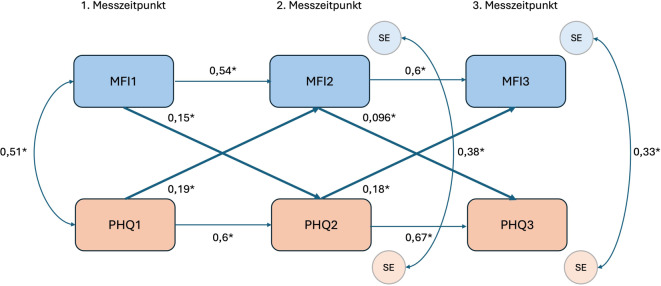
Ergebnis des kreuzlagigen Paneldatenmodells (CLPM) für die Gesamtgruppe (eigene Abbildung). *MFI1‑3* Fatigue zu den jeweiligen Messzeitpunkten, *PHQ* *1–3* Depressivität und Ängstlichkeit zu den jeweiligen Messzeitpunkten, *SE* Standardfehler, *Sternchen* signifikante Koeffizienten

In Abb. 3 ist zu sehen, welche Effekte mit der standardisierten Effektstärke β in der Gesamtgruppe vorliegen. Die autoregressiven Effekte zeigen, dass die Fatigue-Symptomatik sowie Depressivität und Ängstlichkeit größtenteils konstant bleiben und der nächste Messzeitpunkt durch den vorherigen vorhergesagt wird. Die Effekte zwischen dem zweiten und dritten Messzeitpunkt (MFI: $\upbeta$= 0,6 [Konfidenzintervall (KI) 95 %: 0,55–0,65]; *p* < 0,001; PHQ: $\upbeta$= 0,67 (KI: 0,63–0,72); *p* < 0,001) sind tendenziell etwas stärker als die Effekte zwischen dem ersten und zweiten Messzeitpunkt (MFI: $\upbeta$= 0,54 (KI: 0,49–0,59); *p* < 0,001; PHQ: β = 0,6 (KI: 0,55–0,65); *p* < 0,001), jedoch nicht signifikant unterschiedlich. Durch die Kovarianz (cov) von cov(PHQ 1, MFI1) = 0,51 (KI: 0,46–0,56; *p* < 0,001) wird sichtbar, dass es einen querschnittlichen Zusammenhang zwischen Fatigue und Depressivität/Ängstlichkeit gibt. Die kreuzlagigen Effekte MFI1 ⇨ PHQ 2 (β = 0,15 (KI: 0,10–0,21); *p* < 0,001) und PHQ 1 ⇨ MFI2 (β = 0,19 (KI: 0,14–0,25); *p* < 0,001) können als starke Effekte angesehen werden, jedoch unterscheiden sich diese beiden Effekte nicht signifikant voneinander (X^2^(1) = 0,79; *p* = 0,376). Ein ähnliches Bild zeigt sich bei den kreuzlagigen Effekten zwischen dem zweiten und dritten Messzeitpunkt. Hier liegt ein mittlerer Effekt bei MFI2 ⇨ PHQ 3 ($\upbeta$= 0,096 (KI: 0,04–0,15); *p* = 0,001) und weiterhin ein starker Effekt bei PHQ 2 ⇨ MFI3 (β = 0,18 (KI: 0,13–0,24); *p* < 0,001) vor. Auch diese Effekte unterscheiden sich nicht signifikant voneinander (X^2^(1) = 3,79; *p* = 0,051). Anhand der Kovarianzen zwischen den Standardfehlern (SE) zum zweiten (cov (SE (MFI2), SE (PHQ 2)) = 0,38 (KI: 0,33–0,44); *p* < 0,001) und dritten Messzeitpunkt (cov (SE (MFI3), SE (PHQ 3)) = 0,33 (KI: 0,27–0,39); *p* < 0,001) lässt sich weiterhin ein Zusammenhang erkennen, der darauf hinweist, dass Variablen außerhalb des Modells einen Einfluss auf die beiden Variablen haben.

Die Modellgüte liegt insgesamt bei R^2^ = 61,49 %, sodass 61,49 % der Varianz der Outcomes durch die ins Modell aufgenommenen Variablen erklärt wird. Zum zweiten Messzeitpunkt ist die Modellgüte zur Erklärung der Fatigue-Werte (R^2^ = 43,71 %) und der Depressivitäts- und Ängstlichkeitswerte (R^2^ = 47,36 %) etwas schwächer als zum dritten Messzeitpunkt (MFI3: R^2^ = 52,67 %; PHQ 3: R^2^ = 53,83 %).

### Vergleich der Modelle für Personen mit und ohne lang anhaltende Symptome

**Abb. 4:**
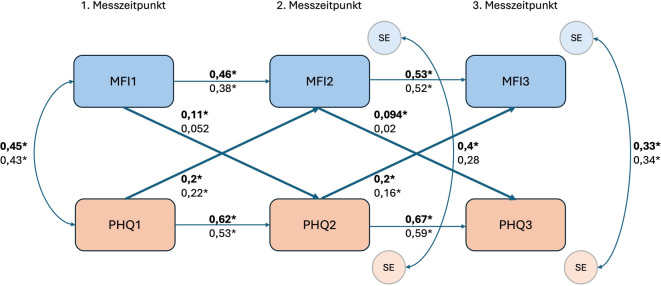
Ergebnis des kreuzlagigen Paneldatenmodells (CLPM) stratifiziert nach Gruppen mit Post-Covid-Syndrom (PCS, fett gedruckt) und ohne PCS (eigene Abbildung). *MFI1–3* Fatigue zu den jeweiligen Messzeitpunkten, *PHQ* *1–3* Depressivität und Ängstlichkeit zu den jeweiligen Messzeitpunkten, *SE* Standardfehler, *Sternchen* signifikante Koeffizienten

In Abb. 4 ist das Modell stratifiziert dargestellt. Bei der Gruppe mit PCS-Symptomen zum zweiten Messzeitpunkt lässt sich beobachten, dass die autoregressiven Effekte des PHQ vergleichbar sind mit dem Gesamtmodell, diese Effekte im MFI jedoch abweichen (MFI1 ⇨ MFI2: $\upbeta$= 0,46 (KI: 0,39–0,53); *p* < 0,001; MFI2 ⇨ MFI3: β = 0,53 (KI: 0,46–0,59); *p* < 0,001). Die querschnittlichen Kovarianzen zwischen den exogenen Variablen MFI1 und PHQ 1 sowie zwischen den jeweiligen Standardfehlern zu den beiden späteren Messzeitpunkten bleiben vergleichbar hoch. In den kreuzlagigen Effekten gibt es jedoch Veränderungen: Der Effekt von Depressivität und Ängstlichkeit auf die Fatigue-Werte ist bei beiden Messzeitpunkten genau gleich (PHQ 1 ⇨ MFI2 & PHQ 2 ⇨ MFI3: β = 0,2 (KI: 0,13–0,27); *p* < 0,001) und größer als im nichtstratifizierten Modell. Die Effekte von Fatigue auf die Depressivität und Ängstlichkeit sind zwar weiterhin vorhanden, jedoch erreichen beide hier eine mittlere Effektstärke (MFI1 ⇨ PHQ 2: $\upbeta$= 0,11 (KI: 0,04–0,17); *p* = 0,002; MFI2 ⇨ PHQ 3: β = 0,09 (KI: 0,03–0,16); *p* = 0,005). Es ist auch hier so, dass es keine signifikanten Unterschiede zwischen den beiden kreuzlagigen Pfaden gibt (2. Messzeitpunkt: X^2^(1) = 2,75; *p* = 0,097; 3. Messzeitpunkt: X^2^(1) = 3,81; *p* = 0,051). Die Modellgüte ist in dieser Gruppe mit R^2^ = 55,87 % etwas schwächer als in der Gesamtauswertung. Weiterhin ist es so, dass die Vorhersagekraft für die Variablen zum dritten Messzeitpunkt (MFI3: R^2^ = 43,24 %; PHQ 3: R^2^ = 52,80 %) stärker ist als zum zweiten Messzeitpunkt (MFI2: R^2^ = 33,39 %; PHQ 2: R^2^ = 45,07 %).

Hinsichtlich der Gruppe ohne PCS zum zweiten Messzeitpunkt (Abb. 4) lassen sich ähnliche Effekte beobachten. Die autoregressiven Effekte bei den Fatigue-Werten zeigen auch hier schwächere Effekte als im Modell der Gesamtgruppe (MFI1 ⇨ MFI2: $\upbeta$= 0,38 (KI: 0,29–0,49); *p* < 0,001; MFI2 ⇨ MFI3: $\upbeta$= 0,52 (KI: 0,43–0,62); *p* < 0,001). Hier sind jedoch auch die autoregressiven Effekte der mit dem PHQ‑4 gemessenen Werte weniger konsistent über die Zeit (PHQ 1 ⇨ PHQ 2: $\upbeta$= 0,53 (KI: 0,43–0,63); *p* < 0,001); (PHQ 2 ⇨ PHQ 3: β = 0,59 (KI: 0,50–0,58); *p* < 0,001). Die gemessenen Kovarianzen sind den vorher genannten sehr ähnlich. Ein Unterschied ergibt sich allerdings durch die Betrachtung der kreuzlagigen Pfade. Während die Effekte von Depressivität und Ängstlichkeit auf die Fatigue-Symptomatik weiterhin signifikant und ähnlich stark wie in der Gesamtgruppe und der Gruppe mit PCS-Symptomen sind (PHQ 1 ⇨ MFI2: β = 0,22 (KI: 0,10–0,34); *p* < 0,001; PHQ 2 ⇨ MFI3: β = 0,16 (KI: 0,05–0,27); *p* = 0,005), sind die Effekte der Fatigue auf die Depressivitäts- und Ängstlichkeitssymptomatik in dieser Subgruppe nicht signifikant (MFI1 ⇨ PHQ 2: β = 0,05 (KI: −0,06–0,17); *p* = 0,372; MFI2 ⇨ PHQ 3: β = 0,02 (KI: −0,09–0,13); *p* = 0,722). Dennoch lassen sich auch hier keine signifikanten Unterschiede zwischen den kreuzlagigen Pfaden feststellen (2. Messzeitpunkt: X^2^(1) = 3,57; *p* = 0,059; 3. Messzeitpunkt: X^2^(1) = 2,55; *p* = 0,11).

Für diese Gruppe ergibt sich insgesamt eine schwächere Modellgüte von R^2^ = 42,61 %, die sich auch in der erklärten Gesamtvarianz für die Fatigue-Werte (MFI2: R^2^ = 26,56 %; MFI3: R^2^ = 36,86 %) und die Depressivität und Ängstlichkeit (PHQ 2: R^2^ = 30,81 %; PHQ 3: R^2^ = 36,01 %) widerspiegelt.

## Diskussion

In der vorliegenden Stichprobe zeigt sich ein konstant erhöhter Anteil an PCS-Betroffenen, der sich über die Zeit von Februar 2021 und März 2022 um 8,5 % verringert. In dieser Gruppe wurden deutlich häufiger klinisch auffällige Werte für Fatigue und Depressivität/Ängstlichkeit festgestellt als in der Gruppe der Personen, die nicht von weiterhin anhaltenden Symptomen berichteten. Auch die maximal gemessenen Werte unterscheiden sich teilweise stark zwischen diesen beiden Gruppen.

Durch die Analysen des CLPM wird deutlich, dass Fatigue und Depressivität und Ängstlichkeit über den zeitlichen Verlauf eine hohe Konstanz aufweisen, d. h., es gibt wenig Schwankungen in der jeweiligen Symptomatik, was sich auch in den gemessenen Mittelwerten widerspiegelt. Die Kovarianz zwischen MFI und PHQ‑4 zeigt, dass es bereits einen querschnittlichen Zusammenhang zwischen den beiden Variablen zum ersten Messzeitpunkt gibt. Dies kann sowohl zu diesem als auch zu den anderen Messzeitpunkten von anderen Variablen außerhalb des Modells beeinflusst sein, wie auch durch die Kovarianzen der Standardfehler zum zweiten und dritten Messzeitpunkt sichtbar wird.

Die kreuzlagigen Pfade zeigen, dass die Fatigue-Symptomatik durch die Depressivitäts- und Ängstlichkeitssymptomatik beeinflusst wird, jedoch auch, dass sich die Fatigue-Werte zur Vorhersage zukünftiger Depressivitäts- und Ängstlichkeitswerte eignen. Da keine signifikanten Unterschiede zwischen den jeweiligen kreuzlagigen Pfaden festgestellt wurden, ist keiner der beiden Effekte kausal.

Die Stratifizierung nach Vorliegen von PCS zum zweiten Messzeitpunkt hebt hervor, dass die zeitlichen Effekte der gemessenen Fatigue in beiden Gruppen und die der gemessenen Depressivität und Ängstlichkeit in der Gruppe ohne PCS schwächer sind als in der Gesamtgruppe. Das Modell hat für die Gruppe mit PCS eine bessere Vorhersagekraft als für diejenige ohne Symptome. Dies äußert sich auch darin, dass bei den Personen, die keine PCS-Symptome haben, keine signifikanten Effekte von den Fatigue-Werten auf die Depressivität und Ängstlichkeit gemessen werden konnten.

Die Ergebnisse stimmen größtenteils mit dem bisherigen Forschungsstand überein, dass ein großer Anteil der ehemals Infizierten weiterhin unter Symptomen leidet, die auch über einen längeren Zeitraum anhalten [5, 6]. Es zeigt sich auch, dass erhöhte Fatigue-Werte bei PCS-Betroffenen deutlich häufiger vorkommen [7, 20], jedoch kann keine klare Trennung erfolgen, welche Auswirkungen ein weiteres Symptom von PCS sind und welche wiederum Folgesymptome der Fatigue sind. Durch die Abschwächung der meisten Effekte in den beiden Subgruppen zeigt sich, dass eine vorliegende PCS-Symptomatik auch als Confounder in dem Modell wirkt. Um dazu geeignete Analysen durchzuführen, wären Messungen mit kleineren zeitlichen Abständen möglicherweise hilfreich, da so die Entstehung der einzelnen Symptome besser nachzuverfolgen wäre.

Im Einklang mit bisheriger Evidenz, zeigt sich durch die quer- und längsschnittlichen Effekte eine wechselseitige Beziehung zwischen Fatigue und psychischen Auffälligkeiten [12, 20]. Diese Beziehung zwischen den Variablen macht deutlich, dass es insbesondere bei lang anhaltendem PCS wichtig ist, der Fatigue-Symptomatik nicht nur mit physischen Behandlungen zu begegnen, sondern gleichzeitig auf die psychischen Folgen dieser Erkrankung einzugehen, wie es auch in dem systematischen Review von Décary et al. [17] herausgestellt wurde. Die deutlich höheren Anteile der auffälligen Fatigue sowie Depressivitäts- und Ängstlichkeitswerte in der Gruppe mit anhaltenden Symptomen unterstützen diese Implikation.

### Limitationen

In dieser Längsschnittuntersuchung könnten Selektionseffekte dahin gehend vorliegen, dass insbesondere Personen an der Längsschnittbefragung teilgenommen haben, die einen erhöhten Leidensdruck aufgrund der PCS-Symptomatik verspüren. Zudem könnten auch die angewandten Filter eine wichtige Personengruppe ausgeschlossen haben. Dieses Vorgehen wurde gewählt, weil psychische Vorerkrankungen mit auffälligen Werten im PHQ‑4 einhergehen und auch erhöhte Fatigue-Werte bedingen könnten.

Eine andere Selektion könnte dadurch bedingt sein, dass bei einigen Personen Angaben zu den untersuchten Variablen fehlten, jedoch würde eine Imputation fehlender Werte bei aus 4 Items bestehenden Skalen zu Verzerrungen führen. Hinsichtlich der Instrumente wurde zur Messung der Fatigue-Symptomatik lediglich eine Subskala des MFI verwendet, während die volle 20-Item-Version des Instruments noch weitere Faktoren der Erkrankung und Symptomatik erheben würde. Teilweise sind die Gruppen mit auffälligen bzw. klinisch relevanten Werten sehr klein, jedoch wurden in das CLPM die Variablen MFI und PHQ als metrische Variablen aufgenommen, sodass dies hier nicht zu Verzerrungen führt.

In der Analyse ist zu berücksichtigen, dass keine der ins Modell aufgenommenen Variablen normalverteilt ist und somit die Voraussetzungen nicht vollständig erfüllt sind, was bei einem CLPM jedoch nicht zu starken Verzerrungen führt [26]. Andere Variablen wie erhaltene Rehabilitationsmaßnahmen, Reinfektionen oder andere Vorerkrankungen wurden nicht ins Modell aufgenommen. Diese könnte jedoch auch Einflüsse auf die gemessenen Variablen haben, was durch die Kovarianzen der Standardfehler gezeigt wird.

## Fazit

Insgesamt bietet die vorliegende Auswertung einen guten Überblick darüber, inwiefern Fatigue und Depressivität und Ängstlichkeit zusammenhängen und welche längsschnittlichen Zusammenhänge es gibt. So können nicht nur die Depressivitäts- und Ängstlichkeitswerte zukünftige Fatigue-Werte vorhersagen, sondern auch umgekehrte Effekte vor allem in der Gruppe mit PCS-Symptomen beobachtet werden. Dies impliziert für Behandlungsansätze von CFS/ME und PCS, dass eine Fatigue-Symptomatik auch psychotherapeutische Bestandteile enthalten sein sollten, um gleichzeitig bestehende Depressivität und Ängstlichkeit zur Linderung der Fatigue-Symptomatik zu behandeln bzw. aufgrund von Fatigue entstehenden psychischen Erkrankungen vorzubeugen.
